# Death of Cullen Andrews Battle of St. Louis

**Published:** 1908-05

**Authors:** 


(Communicated.)

### Death of Cullen Andrews Battle of St. Louis.

On the eve of his departure for his Michigan farm, where he had anticipated a season of rest and a little yachting to restore failing health, Cullen Andrews Battle died suddenly in his apartments at Hotel Jefferson at 6:55 a. m. Sunday, March 22. His brother and business associate, Jesse M. Battle, No. 4463 Lindell Boulevard, was with Mr. Battle when he died. Mrs. Battle, who is well known in social and women's club circles, had been resting at French Lick Springs during the week and did not know of the sudden turn in her husband's condition. She returned home that evening.

Cullen A. Battle was president of Battle & Co. Chemists' Corporation, with offices at No. 2001 Locust street, St. Louis, No. 5 Rue de la Paix, Paris, and No. 76 New Bond street, London. His closest associate was his brother, Jesse, who is treasurer of the corporation.

Several months ago Mr. Battle was forced to forego business because of failing health. Wednesday he became suddenly ill in his apartment and a nurse was summoned. His condition was reported improved until 4 o'clock that morning, when messengers were hastily dispatched for his family physician, Dr. W. F. Kier, No. 3609 Lindell Boulevard. Dr. Kier could not be found, and Dr. Joseph Davie, No. 1204 Chambers street, who had also been called, arrived after Mr. Battle's death. Internal hemorrhage caused the prolonged illness and directly resulted in death. The body was sent to the undertaking rooms of Eberle & Keyes, No. 1108 St. Ange Avenue.

Mrs. Battle, wearied of social duties, had been urged by her husband to take a short rest at French Lick and had been gone a week. Messages telling of Mr. Battle's death were sent to friends at French Lick and Mrs. Battle was summoned home, but she was not told of her husband's death until 5 o'clock that afternoon as her train neared St. Louis. She retained composure until she reached Union Station. Immediately on her arrival at the hotel apartments she was prostrated.

The Battles own a country home at Northport, Mich. The country place comprises a large farm and beautiful home on the water front, with a yacht at their disposal. Mr. Battle spent his summers there, usually leaving the city late in May. On account of his ill health he was preparing to make the trip early this year, and as late as Wednesday night he insisted on reading to his brother a treatise on the merits of fresh country eggs and egg culture.

Mr. Battle was a son of the Rev. Dr. Amos Johnson and Margaret Parker Battle. He was born in Hartford county, North Carolina, May 8, 1848, and educated at the Wilson Collegiate Institute at Wilson, N. C. He began life as a telegraph operator and studied law between the calls of the wire. Later he engaged in the drug business and became a manufacturing chemist. He had been in business in St. Louis since October, 1875. Twenty years ago he married Miss Ida Pugh, of Maysville, Ky.

A sister, Mrs. Joseph H. Foy, resides at No. 2717A Sheridan Avenue. Funeral services were held at 2 o'clock the 24th at the residence of Jesse M. Battle, No. 4463 Lindell Boulevard. Burial was in Bellefontaine Cemetery.

He belonged to the Tuscan Lodge, Ascalon Commandery and Alpha Council and was a member of the Glen Echo and Missouri Athletic Clubs.

